# Virological Findings and Treatment Outcomes of Cases That Developed Dolutegravir Resistance in Malawi’s National HIV Treatment Program

**DOI:** 10.3390/v16010029

**Published:** 2023-12-23

**Authors:** Hope Kanise, Joep J. van Oosterhout, Pachawo Bisani, John Songo, Bilaal W. Matola, Chifundo Chipungu, Katherine Simon, Carrie Cox, Mina C. Hosseinipour, Jean-Batiste Sagno, Risa M. Hoffman, Claudia Wallrauch, Sam Phiri, Kim Steegen, Andreas Jahn, Rose Nyirenda, Tom Heller

**Affiliations:** 1 Partners in Hope, Lilongwe P.O. Box 302, Malawi; hope@pihmalawi.com (H.K.); jsongo@pihmalawi.com (J.S.); cchipungu@pihmalawi.com (C.C.); samphiri@pihmalawi.com (S.P.); 2 Division of Infectious Diseases, David Geffen School of Medicine, University of California, Los Angeles, CA 90095, USA; rhoffman@mednet.ucla.edu; 3 The Lighthouse Trust, Lilongwe P.O. Box 106, Malawi; pbisani@lighthouse.org.mw (P.B.); cwallrauch@lighthouse.org.mw (C.W.); theller@lighthouse.org.mw (T.H.); 4 Directorate of HIV, STI and Viral Hepatitis, Ministry of Health, Lilongwe P.O. Box 30377, Malawi; bwilson@hivmw.org (B.W.M.); ajahn@hivmw.org (A.J.); rnyirenda@hivmw.org (R.N.); 5 Baylor College of Medicine Children’s Foundation-Malawi, Lilongwe P.O. Box 110, Malawi; ksimon@tingathe.org (K.S.); ccox@tingathe.org (C.C.); 6 Baylor College of Medicine, Houston, TX 77030, USA; 7 University of North Carolina Project Malawi, Lilongwe Private Bag A-104, Malawi; mina_hosseinipour@med.unc.edu; 8 Department of Medicine, University of North Carolina at Chapel Hill, School of Medicine, Chapel Hill, NC 27599, USA; 9 DREAM, Communion of St. Egidio, Blantyre, P.O. Box 30355, Malawi; sagnojb@gmail.com; 10 School of Global and Public Health, Kamuzu University of Health Sciences, Lilongwe P.O. Box 30184, Malawi; 11 Department of Haematology & Molecular Medicine, National Health Laboratory Service, Johannesburg 2131, South Africa; kim.steegen@nhls.ac.za; 12 Department of Haematology & Molecular Medicine, University of the Witwatersrand, Johannesburg 2017, South Africa; 13 Department of Global Health, University of Washington, Seattle, WA 98195, USA; 14 International Training and Education Center for Health (ITECH), University of Washington, Seattle, WA 98104-2499, USA

**Keywords:** HIV, dolutegravir, drug resistance, mutations, Malawi, antiretroviral therapy, outcomes

## Abstract

Millions of Africans are on dolutegravir-based antiretroviral therapy (ART), but few detailed descriptions of dolutegravir resistance and its clinical management exist. We reviewed HIV drug resistance (HIVDR) testing application forms submitted between June 2019 and October 2022, data from the national HIVDR database, and genotypic test results. We obtained standardized ART outcomes and virological results of cases with dolutegravir resistance, and explored associations with dolutegravir resistance among individuals with successful integrase sequencing. All cases were on two nucleoside reverse transcriptase inhibitors (NRTIs)/dolutegravir, and had confirmed virological failure, generally with prolonged viremia. Among 89 samples with successful integrase sequencing, 24 showed dolutegravir resistance. Dolutegravir resistance-associated mutations included R263K (16/24), E138K (7/24), and G118R (6/24). In multivariable logistic regression analysis, older age and the presence of high-level NRTI resistance were significantly associated with dolutegravir resistance. After treatment modification recommendations, four individuals (17%) with dolutegravir resistance died, one self-discontinued ART, one defaulted, and one transferred out. Of the 17 remaining individuals, 12 had follow-up VL results, and 11 (92%) were <1000 copies/mL. Twenty-four cases with dolutegravir resistance among 89 individuals with confirmed virological failure suggests a considerable prevalence in the Malawi HIV program. Successful management of dolutegravir resistance was possible, but early mortality was high. More research on the management of treatment-experienced individuals with dolutegravir resistance is needed.

## 1. Introduction

The integrase strand transfer inhibitor (INSTI) dolutegravir is an antiretroviral drug with favorable characteristics. It is very effective in controlling HIV infection, has a high genetic barrier to drug resistance, limited potential for drug interactions, is well tolerated compared to other antiretroviral drugs, and is available in affordable, generic, multidrug-tablets, as well as in convenient and palatable pediatric formulations [1]. For these reasons, the World Health Organization (WHO) recommends dolutegravir as a preferred drug for first- and second-line ART regimens for adults and children [2]. Over the last few years, HIV programs in Malawi and other countries in sub-Saharan Africa have transitioned most individuals on ART to dolutegravir-based regimens [3]. 

Dolutegravir resistance was initially reported in individuals who experienced ART failure after exposure to raltegravir, an older integrase inhibitor with a lower genetic resistance barrier [4]. Among individuals who started a dolutegravir-based regimen when ART-naïve, dolutegravir resistance has been described in very few cases [5,6,7]. Based on in vitro observations and small case series, some risk factors for dolutegravir resistance have been suggested, such as infection with a non-B subtype of HIV, high viral load (VL) and low CD4 cell count at the start of the dolutegravir-based regimen, insufficient adherence to ART, and factors reducing dolutegravir drug levels [5]. Despite the huge population on dolutegravir-based regimens in sub-Saharan Africa, few studies have addressed dolutegravir resistance, likely due to the limited availability of HIV drug resistance (HIVDR) testing for individual client management. To our knowledge, reports on risk factors for dolutegravir resistance, as well as on clinical outcomes of individuals with dolutegravir resistance, have not been published from the region. 

Despite poor socio-economic circumstances, Malawi has a successful HIV program. In a 2020–2021 survey, 88% of around 950,000 persons aged 15–49 years living with HIV (PLHIV) knew their status, of whom 98% were on ART, with 97% having a suppressed viral load (VL). More than 98% of persons on ART are on dolutegravir-based regimens [8].

We have previously described a small number of initial cases with dolutegravir resistance from Malawi [9], and now present a larger case series with more details on observed resistance-associated mutations, and with clinical outcomes after recommendations based on HIVDR test results. We also explored factors associated with dolutegravir resistance.

## 2. Methods

### 2.1. Setting

Full transition to dolutegravir-based regimens took place in Malawi’s HIV program between 2019 and 2021. Non-nucleoside Reverse Transcriptase Inhibitor (NNRTI)-based first-line regimens, as well as Protease Inhibitor (PI)-based first-line and second-line regimens, were replaced with two Nucleoside Reverse Transcriptase Inhibitor (NRTI) + dolutegravir regimens for children and adults. This usually happened without available recent VL results or in the presence of non-suppressed VL results [10]. 

National guidelines indicate that all individuals who have been on PI- or dolutegravir-based regimens for at least one year, and who have confirmed virological failure (defined as a VL result ≥ 1000 copies/mL, confirmed after 3 months with a second VL result ≥ 1000 copies/mL and with recent adherence reported as good), are recommended to have a genotypic HIV drug resistance (HIVDR) test to guide switching to next line regimens [11]. Clinicians need to submit standardized HIVDR test application forms to a national committee that approves HIVDR testing, and later provides clinical recommendations based on genotyping results. Approval was based on information that supports guidelines criteria for HIVDR testing, in particular confirmed virological failure and adequate recent adherence [12]. 

### 2.2. Genotypic HIVDR Testing

After approval for HIVDR testing, clients provided dried blood spot (DBS) samples for transport to the National Health Laboratory Service, Johannesburg, South Africa. Here, two DBS spots (75 μL each) were added to 2 mL of lysis buffer for RNA extraction using NucliSENS easyMAG. HIVDR testing was performed using previously validated in-house protocols adapted from Zhou et al. and Van Laethem et al. [13,14]. Partial pol gene sequences were assembled and edited using RECall (British Columbia’s Centre for Excellence in HIV/AIDS Research). Sequences were loaded onto the Stanford HIV database, genotypic resistance system, version 9.0 (https://hivdb.stanford.edu/hivdb/by-sequences/; accessed on 27 October 2023) to generate resistance reports that provide drug resistance levels and -scores. Resistance was defined as a resistance score of ≥15 (low-level resistance), and INSTI and PI mutations were classified as either major or minor, as per Stanford HIV data base recommendations. In case of failed sequencing, clinicians received a communication with a request to call the client back to the clinic to obtain a new sample for HIVDR testing in South Africa. This was often a protracted process and was not always completed.

### 2.3. Data Collection and Analysis

We reviewed HIVDR testing applications from June 2019 through to October 2022, and we included all individuals on dolutegravir-based regimens at time of application for further analysis. Data were extracted from three sources: the national HIVDR database, based at the Lighthouse Trust, Lilongwe, that included all applications from the country; HIVDR test application forms, and genotyping result forms. We contacted treating clinicians in case of missing data, and to obtain information about standardized ART outcomes and VL results for individuals whose sample contained dolutegravir resistance mutations. We used standardized ART outcomes as defined in Malawi HIV management guidelines [11], as follows: Defaulted—not returned to the clinic for at least 2 months after the patient is expected to have run out of ART drugs, and not known to have transferred to another clinic, stopped ART or died; Stopped ART—last known to be alive and known to have stopped taking ART; Died—reliable report about the death; Alive on ART—registered on ART at the clinic and not defaulted, died or stopped ART. We provide details of client characteristics and genotyping results by 3 categories: 1. presence of major dolutegravir resistance-associated mutations; 2. presence of major NRTI, NNRTI and/or PI resistance-associated mutations but no dolutegravir resistance-associated mutations; and 3. wild type (no mutations). For clinical outcomes after implementation of clinical recommendations based on HIVDR results, we categorized VL results according to national guidelines as suppressed (<200 copies/mL), low level viremia (200–999 copies/mL), and high (≥1000 copies/mL). We used logistic regression analysis to explore factors associated with presence of dolutegravir resistance-associated mutations among samples that had undergone successful integrase sequencing. We categorized samples as with or without dolutegravir resistance (≥15 Stanford resistance score) as the outcome variable. We first fitted univariate logistic regression models with the outcome variable against each of the explanatory variables in our dataset to obtain unadjusted odds ratios. To obtain adjusted odds ratios, we then fitted a multivariable logistic regression model where age, sex, high-level NRTI resistance, TB treatment during dolutegravir-based ART, and duration of viremia during dolutegravir exposure were included based on presumed biological plausibility of their association with the outcome. We subsequently used forward stepwise elimination to add/drop the other variables to/from the model. Variables were retained in the model if statistically significant, which was determined with the Wald test. This approach was taken to control for possible confounding in the absence of a formal causal theory guided by literature.

## 3. Results

### 3.1. HIVDR Testing Cascade

**Figure 1:**
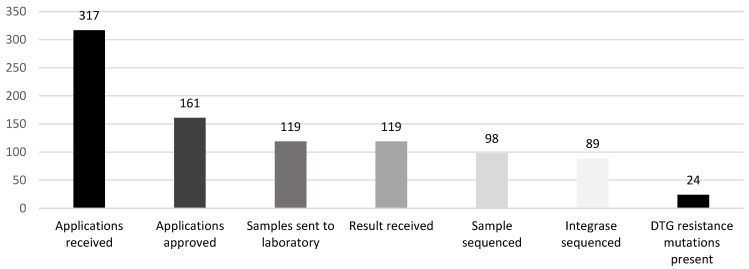
HIV drug resistance testing cascade (July 2019–October 2022), National HIV Program Malawi.

During the study period, 317 applications for HIVDR testing for clients on dolutegravir-based regimens were received. Of these, 51% that fulfilled eligibility criteria for HIVDR testing were approved. Further drop-offs occurred at subsequent steps of the HIVDR testing cascade (Figure 1). No sample was collected in 26% of approved applications. Sequencing failed completely in 21 samples, and of the 98 samples with sequences, integrase gene sequencing failed in nine, meaning that integrase sequencing was successful in 75% of the samples received. Out of 89 integrase sequences that formed the population for further analysis, 24 (27%) had dolutegravir resistance-associated mutations. 

### 3.2. Characteristics of Individuals with Integrase Sequences and Their HIVDR Results

An overview of the client characteristics whose samples underwent successful integrase sequencing is provided in Table 1. The median age was 29 years, and clients with dolutegravir resistance were older than those in the two categories without dolutegravir resistance. At the time of application, all clients were on 2NRTI + dolutegravir regimens. None were on a third line regimen, which in Malawi always contains a PI, generally darunavir. In two of the 89 samples with integrase sequences, the protease (PR) and reverse transcriptase (RT) sequences were missing. All 24 cases with dolutegravir resistance had been on a 2NRTI + NNRTI regimen initially. Ten had been exposed to PIs and none to raltegravir or elvitegravir. Of the 22 samples with dolutegravir resistance mutations that were fully sequenced (i.e., with available PR and RT sequences), 20 (91%) had NRTI resistance mutations, and two (3.4%) PI resistance mutations. In the category “HIVDR mutations present, no DTG resistance”, 20/35 (57%) only had NNRTI mutations, and had no resistance-associated mutations to drugs in their regimen (i.e., 2NRTI + dolutegravir) at the time of application for HIVDR testing. The prevalence of the M184V, the signature lamivudine mutation, was remarkably low in the same category: 8.6% (3/35).

The dolutegravir resistance-associated mutations observed in our cases are summarized in Table 2. There were seven different mutations detected in various combinations. R263K was the most common (67%; 16/24), followed by E138K (28%; 7/24), and G118R (25%; 6/24). Apart from these major dolutegravir resistance associated mutations, we also observed the minor dolutegravir resistance-associated mutations H51Y, L74M, Q95K, G149A, S153SF, and E157Q. Overall, dolutegravir resistance scores indicated low-level resistance in 8% (2/24), intermediate resistance in 54% (13/24), and high-level resistance in 38% (9/24). In eight samples, a combination of three major INSTI mutations was detected; whereas the remaining 16 patients presented with a single integrase inhibitor resistance-associated mutation, including one individual with a single minor dolutegravir resistance associated mutation (S153SF), interpreted as low-level resistance.

### 3.3. Factors Associated with HIVDR Resistance

We compared individual characteristics among clients with and without dolutegravir resistance-associated mutations (any level of resistance). In an unadjusted analysis, male sex and age ≥ 40 years, as well as high-level NNRTI resistance, high-level NRTI resistance, and presence of the M184V mutation were significantly associated with dolutegravir resistance (Table 3). While differences were present between the two groups in duration on ART, duration of viremia while on dolutegravir, year of application, and taking tuberculosis treatment while on dolutegravir (all 3 individuals who developed dolutegravir resistance received double-dosed dolutegravir), these associations were not statistically significant. In the multivariable analysis, only age ≥ 40 years and presence of high-level NRTI resistance remained significantly associated with dolutegravir resistance. The association of TB treatment during dolutegravir-based ART showed borderline significance. 

### 3.4. ART Outcomes of Individuals Diagnosed with Dolutegravir Resistance (Table S1)

Recommended regimens for individuals with dolutegravir resistance, based on HIVDR test result and ART history, included 2NRTI + PI (n = 7), 2NRTI + dolutegravir (double-dose) + PI (n = 14), and 2NRTI + dolutegravir (double-dose) (n = 3). Of 24 individuals with dolutegravir resistance, 4 (17%) died, of whom one death occurred before the HIVDR result became available; 1 self-discontinued ART; 1 defaulted; and 1 transferred to another clinic before the recommended regimen was implemented. Of the remaining 17 clients alive on ART, VL results were available for 12 (71%) at a median of 12 (range 3–17) months after the treatment recommendation. Five clients had not yet undergone VL testing after the recommended change of regimen. Of 12 VL results, 10 (78%) were <200 copies/mL, 1 was low-level viremia (265 copies/mL), and 1 was >1000 (1562) copies/mL. VL results did not appear to differ substantially by recommended regimen or dolutegravir resistance score (Table S1). One of the cases with intermediate dolutegravir resistance (score 30) achieved suppressed VL (<200 copies/mL) despite continuing on the same regimen (TDF/3TC/DTG) due to a miscommunication.

## 4. Discussion

The importance of the emergence of dolutegravir resistance in sub-Saharan Africa cannot be underestimated. In Malawi, around 99% of 940,000 individuals on ART in the Malawi national HIV program were on dolutegravir-based regimens by half 2023 [15]. In this setting, we found 24 cases with dolutegravir resistance among 89 samples (27%) from individuals with confirmed virological failure while on regimens with dolutegravir and 2NRTIs. Given that this was not a representative national survey, that there were large drop-offs in the HIVDR testing cascade (only 28% of applications underwent successful integrase sequencing), and with the prolonged viremia prior to resistance testing in the study population, this likely represents a select population, limiting the generalizability of our prevalence estimate of dolutegravir resistance for Malawi. Few reports on dolutegravir resistance from sub-Saharan African countries have been published. An observational study from 2019 in Chiradzulu District, Malawi included around 1300 participants who had transitioned to dolutegravir in programmatic circumstances [16]. After six months, six individuals had confirmed virological failure, of whom two had dolutegravir resistance. A study of children and adults from Tanzania found 5.8% (8/137) prevalence of dolutegravir resistance in individuals with VL results of ≥1000 copies/mL [17]. In a report from Botswana, 11 cases with INSTI resistance-associated mutations were found in 34 integrase sequences (32.4%), though seven of these had been induced by raltegravir exposure only [18]. In Nigeria, one case of dolutegravir resistance was found among 66 individuals (1.5%) with virological failure on dolutegravir regimens [19]. These reports indicate that persons who transitioned to dolutegravir from mainly NNRTI-based regimens infrequently develop dolutegravir resistance. The observations we present here suggest that dolutegravir resistance may be more common among individuals with confirmed virological failure in Malawi, particularly in individuals with prolonged viremia who continued dolutegravir-based ART. A national survey was completed recently, and will provide more representative and generalizable estimates of dolutegravir resistance prevalence in Malawi. 

We observed seven of the ten mutations that the Stanford database recognizes as major integrase inhibitor resistance mutations [20]. Of interest, S147G, while not recognized as a major dolutegravir resistance mutation, occurred regularly among the cases with dolutegravir resistance (17%), none of whom had been exposed to integrase inhibitors other than dolutegravir, and it induced low-level dolutegravir resistance as a single mutation (case 7). Seven different mutation patterns were observed. 15/24 (63%) cases had a single mutation, including R263K, G118R, and S147G. R263K was by far the most common mutation. A recent global review of emergent dolutegravir resistance mutations suggests that four largely non-overlapping dolutegravir resistance pathways exist that are characterized through mutations at four signature positions: R263K, G118R, N155H, and Q148H/R/K [21]. In our cases, we observed the R263K (15/24) and G118R (5/24) pathways. As previously reported [22], G118R occurred together with R263K in one case, and Q148R with N155H in another, with both combinations resulting in high-level resistance. Based on these observations, we note that subtype C seems to follow a similar genotypic resistance development as indicated in the Stanford database. We also speculate that the variety of mutations found is an indication of progression of dolutegravir resistance in the population on ART.

Some factors that may favor dolutegravir resistance development are prevalent in programmatic circumstances in Malawi, including the predominance of HIV subtype C [9,23] and the occurrence of functional dolutegravir monotherapy. The latter may have occurred frequently, given that transitions to dolutegravir-based regimens often happened without suppressed VL, or without a recent VL result [9]. In such a situation, dolutegravir may be introduced in the presence of extensive resistance to the NRTI backbone. On the other hand, a study that included individuals with long-term virological failure of first-line NNRTI-based ART in resource-limited settings [24], demonstrated that high levels of viral suppression were achieved with dolutegravir-based second-line regimens in the presence of resistance to NRTI backbone drugs. This may question the relevance of functional dolutegravir monotherapy, although this randomized clinical trial may not fully reflect the programmatic circumstances of our cases. In our cases, long delays in high VL management and HIVDR testing procedures generally resulted in long-term HIV replication in the presence of dolutegravir exposure, which may facilitate development and accumulation of dolutegravir resistance mutations. In a multivariable analysis, we observed that older age and presence of high-level NRTI resistance were associated with higher probability of dolutegravir resistance. Similar to our findings, a recent study from Western countries and South Africa, the latter country contributing only 2% of 599 participants with virological failure on dolutegravir-based ART, found that the risk of dolutegravir resistance was increased with presence of NRTI resistance [25]. Investigators from the ADVANCE study in South Africa have proposed that virological failure on a dolutegravir-based regimen may be facilitated by baseline NNRTI resistance [26], but the mechanism for this association is not clear, and virological failure was not caused by dolutegravir resistance. We are not aware of other reports from sub-Saharan Africa that determined factors associated with dolutegravir resistance. 

We have earlier reported one individual (also included in this case series) [9], who was ART naïve and developed dolutegravir resistance on first-line treatment, which happens extremely rarely [5]. However, the presence of NNRTI drug resistance mutations in this case opened the possibility of undisclosed previous ART. 

Four of the 24 cases (17%) with dolutegravir resistance died within one year after HIVDR testing. This underlines that HIVDR needs to be managed diligently to prevent severe morbidity and mortality. However, uncertainties about the management of patients with persistent viremia on dolutegravir-based regimens in settings with limited HIVDR testing capacity remain, including about the optimal duration of adherence support measures, and the timing of genotyping and ART switches [27]. Such decisions may be facilitated by adding a point-of-care (POC) tenofovir urine assay in algorithms for management of individuals with high VL results, to help differentiating between non-adherence and potential resistance [28]. Recently developed POC HIVDR tests may also become beneficial in this regard, if dolutegravir resistance mutations can be demonstrated directly, or if they can detect the M184V mutation, given that 91% of our cases with dolutegravir resistance had M184V, while among cases without dolutegravir M184V prevalence was 5%. However, POC HIVDR tests do not yet have the required characteristics for use in our setting [29]. The high mortality that we observed also points to the need to do routine advanced HIV disease screening among individuals with longer-term virological failure. 

Individuals with dolutegravir resistance who remained on ART had satisfactory virological results (92% with VL < 1000 copies/mL). As publications about ART outcomes after occurrence of dolutegravir resistance are restricted to case reports [30], we cannot compare these results to studies from the region. In cases with low- and intermediate-level dolutegravir resistance, doubling the dose of dolutegravir as the only intervention may be a viable management approach. This has been associated with successful outcomes in ART experienced patients who harbored HIV with integrase inhibitor resistance mutations due to previous treatment with raltegravir or elvitegravir [4]. We only had one case where this approach was implemented and a VL result was available. While that led to VL suppression, it remains uncertain if this strategy is as successful as switching to a PI-based regimen, especially with higher dolutegravir resistance scores. More research from sub-Saharan Africa is needed to determine optimal management approaches for treatment-experienced persons with dolutegravir resistance.

Our study benefited from a substantial number of cases with dolutegravir resistance in real-life, programmatic circumstances. We also point to some limitations, including the non-representativeness of the study sample, as well as the low power of the analysis of factors associated with dolutegravir resistance. Therefore, these results should be confirmed in larger studies. Routine CD4 count monitoring is not recommended in national guidelines, and CD4 testing was not available in many clinics. Therefore, we did not have CD4 count results for most cases at the time of application nor at transition to dolutegravir-based ART. We also had very few VL results at the start of dolutegravir-based ART and therefore could not assess the importance of low CD4 and high VL as drivers of dolutegravir resistance. We did not include adherence data, however approval for HIVDR testing was granted only if recent good adherence had been documented with pill counts and self-report on application forms. Duration of follow up after treatment switch recommendations was variable and in some cases short. Lastly, follow-up virological results were not available for five individuals with dolutegravir resistance due to delayed VL sampling at the health facility.

## 5. Conclusions

We observed dolutegravir resistance in 24 out of 89 (27%) successfully sequenced samples from individuals with confirmed virological failure in the routine setting of the Malawi HIV program. This raises concern that prevalence of dolutegravir resistance may be considerable, particularly with prolonged viremia. Seven out of ten recognized major integrase inhibitor resistance mutations have now been found in Malawi, and specific mutation patterns were recognized. Successful management of dolutegravir resistance was possible, but early mortality was high. These findings indicate the need for a better and more expedient capacity to distinguish dolutegravir resistance from poor adherence in our setting. In addition, more research is needed to determine optimal ART management of treatment-experienced individuals harbouring dolutegravir resistance. 

## Figures and Tables

**Table 1:** Characteristics of clients with samples that underwent successful integrase sequencing and HIVDR result summaries.

Variable	Samples with Integrase Sequences		DTG Resistance Mutations Present		Wild Type		HIVDR Mutations Present, No DTG Resistance Mutations	
n	89		24		30		35	
	n	%	n	%	n	%	n	%
Presence high level NNRTI resistance #								
No	35	40.7	4	18.2	30	100.0	1	2.9
Yes	51	59.3	18	81.8	0	0.0	33	97.1
Presence of high level NRTI resistance *								
No	69	80.2	11	50.0	30	100.0	28	82.4
Yes	17	19.8	11	50.0	0	0.0	6	17.6
Presence of M184V mutation								
No	63	74.3	2	9.1	30	100.0	31	91.2
Yes	23	26.7	20	90.9	0	0.0	3	8.8
Median age (range)	29 (4–63)		40 (5–63)		26 (11–56)		20 (4–54)	
age 0–9	5	5.6	2	8.3	0	0.0	3	8.6
age 10–19	32	36.0	4	16.7	14	46.7	14	40.0
age 20–49	42	47.2	14	58.3	13	43.3	15	42.9
age 50+	10	11.2	4	16.7	3	10.0	3	8.6
Sex								
Female	51	57.3	9	37.5	20	66.7	22	62.9
Male	38	42.7	15	62.5	10	33.3	13	37.1
ART regimen at time of resistance testing								
TDF/3TC/DTG	62	69.7	16	66.7	23	65.7	23	63.6
AZT/3TC + DTG	9	10.1	2	8.3	2	6.7	3	13.6
ABC/3TC + DTG	16	18.0	6	25.0	4	13.3	6	17.1
pABC/3TC + DTG	1	1.1	0	0.0	1	3.3	0	0.0
pABC/3TC + pDTG	1	1.1	0	0.0	0	0.0	1	2.9
Median duration ART, months (range)	87 (14–231)		97 (20–202)		53 (14–231)		92 (27–216)	
ART 0–36 months	17	19.1	3	12.5	7	23.3	3	8.6
ART 37–72 months	20	22.5	4	16.7	12	40.0	6	17.1
ART 73+ months	52	58.4	17	70.8	11	36.7	26	74.3
Median duration on DTG, months (range)	22 (5–56)		24 (8–46)		22 (8–51)		20 (5–56)	
DTG 0–12 months	18	20.2	5	20.8	5	16.7	8	22.9
DTG 13–24 months	36	40.4	7	29.2	14	46.7	15	42.9
DTG 25+ months	35	39.3	12	50.0	11	36.7	12	34.3
Median duration viremia on DTG, months (range)	12 (0–35)		14 (0–31)		13 (2–35)		9 (0–28)	
0–12 months	50	56.2	10	41.7	15	50.0	25	71.4
13+ months	39	43.8	14	58.3	15	50.0	10	28.6
TB treatment during DTG regimen								
No	82	92.1	21	87.5	28	93.3	33	94.3
Yes	7	7.9	3	12.5	2	6.7	2	5.7
Application year								
2020	2	2.2	0	0.0	2	6.7	0	0.0
2021	52	58.4	12	50.0	18	60.0	22	62.9
2022	35	39.3	12	50.0	10	33.3	13	37.1

# High level is based on Stanford HIVDR dB version 9.0, HIVDR resistance score ≥ 60. * High level resistance to at least one of TDF, AZT, ABC. TDF, tenofovir disoproxil; 3TC, lamivudine; DTG, dolutegravir, AZT, zidovudine; ABC, abacavir; pABC, pediatric ABC; ART, antiretroviral therapy; NRTI, nucleoside reverse transcriptase inhibitor; NNRTI, non-NRTI.

**Table 2:** Observed dolutegravir resistance associated mutations ^$^ with resistance score and level ^†^.

Case ^#^	R263K	E138K	S147G ^	T66A/I	G118R	Q148R	N155H	Stanford Resistance Score (Level)
1	x							40 (intermediate)
2	x							30 (intermediate)
3	x	x	x					60 (high)
4	x							30 (intermediate)
5	x							40 (intermediate)
6	x							30 (intermediate)
7			x					20 (low)
8	x							30 (intermediate)
9	x							30 (intermediate)
10		x		x	x			70 (high)
11	x							30 (intermediate)
12 *								15 (low)
13		x		x	x			80 (high)
14					x			60 (high)
15		x		x	x			85 (high)
16	x	x	x					60 (high)
17	x							30 (intermediate)
18	x							30 (intermediate)
19			x			x	x	125 (high)
20	x							50 (intermediate)
21		x		x	x			80 (high)
22	x							30 (intermediate)
23	x							30 (intermediate)
24	x	x			x			110 (high)

$ Major INSTI mutations E92, G140 and Y143 were not observed during the study period (Y143 mutations do not affect dolutegravir susceptibility). † Based on Stanford HIVDR dB version 9.0. # Cases are listed in chronological order of the application date. ^ S147G is not considered a major dolutegravir resistance-associated mutation, but was common among our clients who had not been exposed to integrase inhibitors other than dolutegravir and as a single mutation induced low-level resistance. * The minor dolutegravir resistance associated mutation S153SF classified the dolutegravir resistance level in this sample as low.

**Table 3:** Factors associated with dolutegravir resistance among samples from individuals with confirmed virological failure and successful integrase sequencing.

Variable			Unadjusted ORs for DTG Resistance				Adjusted ORs for DTG Resistance			
	DTG Resistance (n = 24)	No DTG Resistance (n = 65)	OR	95% CI		*p*-Value	OR	95% CI		*p*-Value
Age categories, years (%)										
0–19	25.0	47.7	1 (ref)				1 (ref)			
20–39	16.7	27.7	1.15	0.29	4.62	0.846	2.63	0.42	16.6	0.305
40+	58.3	24.6	4.52	1.46	14.0	0.009	4.77	1.13	20.1	0.033
Male sex (%)	62.5	35.4	3.04	1.15	8.03	0.025	2.72	0.69	10.8	0.16
TDF/3TC (%) vs. any other (AZT/3TC & ABC/3TC) backbone	66.7	70.8	0.83	0.30	2.25	0.709				
Median duration on ART (months)	97.0	83.0	1.01	1.00	1.01	0.299				
Median duration on DTG (months)	24.0	21.0	1.02	0.97	1.06	0.452				
Median duration viremia on DTG, months (%)										
0–12	41.7	61.5	1 (ref)				1 (ref)			
13+	58.3	38.5	2.24	0.86	5.81	0.097	1.66	0.50	5.54	0.41
TB treatment during DTG regimen (%)	12.5	6.2	2.18	0.45	10.6	0.333	5.04	0.79	32.0	0.09
Application year 2022 (%) vs. 2020 or 2021	50.0	35.4	1.83	0.71	4.71	0.213				
Presence high level NNRTI (NVP and/or EFV) resistance (%)	81.8	51.6	4.23	1.29	13.9	0.018				
Presence high level NRTI (TDF, AZT and/or ABC) resistance (%)	50.0	9.4	9.67	2.96	31.6	0.000	10.0	2.57	39.1	0.00
Presence of M184V mutation (%)	90.9	4.7	203	31.7	1305	0.000				

OR, odds ratio; CI, confidence interval; TDF, tenofovir disoproxil; 3TC, lamivudine; DTG, dolutegravir, AZT, zidovudine; ABC, abacavir; ART, antiretroviral therapy; NVP, nevirapine; EFV, efavirenz; NRTI, nucleoside reverse transcriptase inhibitor; NNRTI, non-NRTI.

## Data Availability

The data are owned by the Ministry of Health. Requests for review of the data can be submitted to Bilaal W Malata, Ministry of Health, Department of HIV-AIDS, Lilongwe, Malawi. bwilson@hivmw.org.

## Supplementary Materials

The following supporting information can be downloaded at: https://www.mdpi.com/article/10.3390/v16010029/s1, Table S1: Standardized ART outcomes and viral load results of individuals with dolutegravir resistance.
