# Supplementary material for: Virological Findings and Treatment Outcomes of Cases That Developed Dolutegravir Resistance in Malawi’s National HIV Treatment Program
Source: Viruses. 2023 Dec 23;16(1):29. doi: 10.3390/v16010029 (PMC10819735; doi:10.3390/v16010029)
Supplement: Supplementary file 1 [file viruses-16-00029-s001.zip › viruses-2727951-supplementary.pdf]

Table S1. Standardized ART outcomes and viral load results of individuals with dolutegravir resistance

Application year	Age	Sex	DTG resistance score	Recommended regimen**	Date implemented	Date last VL result	time recommendation to VL	VL result	ART outcome	ART outcome date
2021	40	Male	60	TDF/3TC/DTG+DRV/r	NI	NA	NA	NA	LTFU	May-21
2021	42	Male	30	TDF/3TC/DTG+DRV/r	Jun-21	Nov-22	12	LDL	Alive in Care	
2021	43	Male	20	TDF/3TC/DTG+DTG	NI	Feb-22	13	175,923*	TO	May-22
2021	43	Female	30	TDF/3TC/DTG + DTG + DRV/r	NI	Jan-2023	14	<40	Alive in care	
2021	47	Male	30	TDF/3TC/DTG + DTG + DRV/r	NI	not drawn	NA	NA	Died	Jun-22
2021	48	Male	30	None	NA	NA	NA	NA	Died	Jul-21
2021	14	Male	30	TDF/3TC + ATV/r	Jan-21	June-2022	17	LDL	Alive in Care	
2021	40	Male	40	TDF/3TC/DTG+DRV/r	Nov-23	May-2023	6	1,562	Alive in Care	
2021	15	Female	30	TDF+3TC+DTG+DTG+DRV/r	Jun-21	Apr-22	9	97	Alive in Care	
2021	20	Male	40	TDF+3TC+DTG+DTG+DRV/r	Sep-22	not drawn	NA	NA	Died	Dec-22
2021	41	Male	105	TDF+3TC+DTG+DTG+DRV/r	Nov-21	Jul-22	13	57	Alive in Care	
2021	62	Female	70	TDF+3TC+DTG+DTG+DRV/r	NI	Mar-2022	unknown	259,000	Stopped ART	Mar-22
2022	40	Male	50	AZT/3TC+ATV/r	Mar-23	not drawn	NA	NA	Alive in care	
2022	30	Female	85	TDF/3TC+ATV/r	Sep-22	Apr-23	8	LDL	Alive in Care	
2022	7	Female	15	ABC/3TC+DTG+DTG	Sep-22	Sep-23	12	<40	Alive in Care	
2022	21	Female	30	TDF/3TC/DTG+DRV	Jan 2023	Mar-23	3	<40	Alive in Care	
2022	55	Male	60	TDF/3TC/DTG+DRV/r	Sep-22	not drawn	NA	NA	Died	Apr-23
2022	46	Male	60	TDF/3TC+ATV/r	Sep-22	Sep-23	12	<40	Alive in Care	
2022	17	Male	80	TDF/3TC+ATV/r	Mar-23	not drawn	NA	NA	Alive in Care	
2022	12	Female	30	ABC/3TC+DTG+DTG	Mar-23	not drawn	NA	NA	Alive in Care	
2022	5	Female	30	ABC/3TC+DTG+DTG	May-22	Jun-23	13	97	Alive in Care	
2022	63	Male	30	AZT/3TC + ATV/r	Feb-23	not drawn	NA	NA	Alive in Care	
2022	34	Female	110	2NRTIs + DTG + DRV/r	Jul-23	not drawn	NA	NA	Alive in Care	
2022	52	Male	125	TDF/3TC + ATV/r	Nov-22	Feb-23	4	265	Alive in Care	

*Sample taken before treatment change. ** Recommended regimens were based on genotyping results and previous ART history. 10/24 cases had previous protease inhibitor exposure. At the start of dolutegravir-based ART, one case was ART naïve, 4 cases had 1 previous regimen, 10 had two previous regimens, 7 had three and 1 had four previous regimens.
